# Allostatic load amplifies the effect of blood lead levels on elevated blood pressure among middle-aged U.S. adults: a cross-sectional study

**DOI:** 10.1186/1476-069X-12-64

**Published:** 2013-08-16

**Authors:** Ami R Zota, Edmond D Shenassa, Rachel Morello-Frosch

**Affiliations:** 1 Program on Reproductive Health and the Environment, University of California, San Francisco, Oakland, CA, USA; 2 Department of Environmental and Occupational Health, School of Public Health and Health Services, George Washington University, Washington, DC, USA; 3 Maternal and Child Health Program and Department of Epidemiology and Biostatistics, University of Maryland, College Park, Maryland, USA; 4 Department of Environmental Science, Policy and Management and School of Public Health, University of California, Berkeley, California, USA

**Keywords:** Blood pressure, Cumulative impacts, Environmental health, Lead, Stress

## Abstract

**Background:**

Scientists and regulators have sought to understand whether and how physiologic dysregulation due to chronic stress exposure may enhance vulnerability to the adverse health effects of toxicant exposures. We conducted a cross-sectional study to determine whether allostatic load (AL), a composite measure of physiologic response to chronic exposure to stress, amplifies the effect of lead exposure on blood pressure among middle-aged adults.

**Methods:**

We analyzed associations between blood lead levels and blood pressure in a nationally representative sample of 8,194 U.S. adults (aged 40-65 years) participating in the National Health and Nutritional Examination Survey, 1999--2008. Outcomes were elevated systolic (≥ 140 mm Hg) and diastolic (≥ 90 mm Hg) blood pressure. AL was defined as the aggregate score of seven components, reflecting dysregulation of the cardiovascular, inflammatory, and endocrine systems.

**Results:**

Logistic regression models showed a linear dose-response relationship for quintiles of blood lead and elevated systolic blood pressure in the high AL group (p = 0.03) but not the low AL group (p = 0.24). Similarly, the relationship between lead exposure and elevated diastolic blood pressure was stronger among the high AL group than the low AL group. Within the high AL group, the fourth and fifth quintiles had significantly elevated odds of elevated blood pressure compared to lowest quintile [OR = 1.92, (95% CI, 1.07, 3.47) and OR =2.28 (95% CI, 1.33, 3.91), respectively]. In the low AL group, none of the quintile effects were significantly different than the referent group although there was evidence of a linear trend (p =0.03). The lead by AL interaction term was not statistically significant for either systolic or diastolic blood pressure models.

**Conclusions:**

Results suggest that higher AL may amplify the adverse effects of lead on blood pressure. Future research should assess the implications of cumulative exposures to environmental and social stressors for regulatory decision-making.

## Background

Regulatory agencies and environmental health scientists are beginning to examine whether and how chronic stress exposure potentially amplifies human vulnerability to the adverse health effects of toxicant exposures [1]. Emerging evidence suggests that cumulative physiological “wear and tear” resulting from chronic over-activity of the body’s stress-response system may impair immune functioning and increase vulnerability to environmental stressors [2] by increasing the absorption of toxicants into the body through increased respiration, perspiration, and consumption [3]; compromising the body’s defense systems against toxicants; affecting the same physiological processes as environmental agents; and directly causing illness [4,5].

High blood pressure is a common condition among U.S. adults and a major risk factor for strokes, heart attacks, congestive heart failure, and kidney disease [6,7]. Minority and lower socio-economic status (SES) populations are at elevated risk of hypertension [6]. These populations also encounter the double jeopardy of elevated exposures to environmental hazards combined with chronic social stressors [8,9]. While the root causes of hypertension remain poorly understood, growing evidence indicates that the interaction of environmental, social, and genetic factors may partially explain the persistent racial and socioeconomic disparities in cardiovascular outcomes, such as hypertension [10-12].

Lead is a ubiquitous environmental contaminant that can impair the cardiovascular system. Although lead was removed from gasoline and household paint in the 1970s, the general population, and low SES populations in particular, continue to be exposed to lead through occupational exposures, lead-based paint in older housing stock, and the gradual release of lead from bone, which serves as the body’s principal, long term repository for this pollutant [13]. Cross-sectional and longitudinal epidemiologic studies demonstrate associations between lead exposure and elevated blood pressure including development of hypertension [14-17]. Animal studies have confirmed this relationship and elucidated mechanisms by which lead may influence blood pressure regulation [18]. A systematic review by Navas-Acien et al. [19] concluded that the evidence was sufficient to infer a causal relationship between lead exposure and hypertension.

Chronic stress has also been associated with hypertension [20] and may interact with lead to stimulate the hypothalamic–pituitary–adrenal (HPA) axis, promote oxidative stress and inflammation, augment adrenergic activity, and alter the renin-angiotensin system [18,21,22], which are critical to blood pressure regulation. Chronic stress may result from myriad experiences such as job stress, racial discrimination, and place-based stressors such as lack of basic services, and exposure to violence [23-26]. Allostasis refers to how the body’s stress-response systems regulate internal physiology in response to chronic exposure to physical, social, and environmental stressors [27]. Allostatic load (AL) refers to the cumulative biological burden exacted by ongoing dysregulation of the stress-response due to chronic stress exposure [2,27,28]. AL encompasses physiologic dysregulation of multiple systems affected by over-activation of the HPA axis including, but not limited to, the central nervous system, cardiovascular, immune, inflammatory, and endocrine systems [29]. Previous studies have linked higher AL scores with increased risk of aging-related health outcomes such as incident cardiovascular disease, decline in physical functioning, decline in cognitive function, and mortality [30-32]. Therefore, allostatic load is a composite biomarker of the cumulative biological burden exacted by ongoing disruption of the body’s stress-response system [33] that may increase vulnerability to the adverse health effects of toxicants, such as lead [8].

While potential synergistic effects between chronic stress and lead exposures on blood pressure are biologically plausible, only one epidemiologic study has examined these interactions. Peters et al. [34] reported a significant interaction between bone lead exposures and self-reported measures of stress on the development of hypertension among a cohort of older, white men in the Boston area. However, no previous study has examined whether AL amplifies vulnerability to the adverse health effects of exposures to environmental toxicants, in particular, lead. We hypothesized that middle-aged adults with higher AL would be more vulnerable to the lead-induced effects on blood pressure than adults with lower AL. To test this hypothesis, we evaluated the association between blood lead level and elevated systolic and diastolic blood pressure in a cross-sectional study of middle-aged men and women from the United States (U.S.). We then stratified our sample between high versus low AL to examine differences in effect estimates between these two groups.

## Methods

### Study population

The National Health and Nutritional Examination Survey (NHANES), conducted by the Centers for Disease Control and Prevention (CDC), are a nationally representative survey and physical examination of the health and nutritional status of the civilian, non-institutionalized U.S. population. The survey also includes measurement of environmental chemicals in blood and urine (Further information at http://www.cdc.gov/nchs/nhanes.htm). NHANES obtained informed consent from all study participants. Due to the complex survey design, separate sample weights are assigned to each survey participant; each participant represents approximately 50 000 other U.S. residents.

The present study combines participants from five cycles of NHANES, spanning the years 1999 to 2008. African Americans, Mexican Americans, and low-income persons were oversampled in these survey cycles [35]. In this study, we restricted the study population to the 9918 participants who were 40-65 years of age to minimize the effect of confounding by age, which is strongly associated with lead exposure, blood pressure, and AL [7,14,36]. Of these, we excluded participants who were missing measurements of blood lead, blood pressure, components of AL, any model covariates or who were currently pregnant (N = 1724), leaving a total of 8194 eligible participants for our analyses.

### Blood pressure outcomes

Three consecutive measurements of systolic and diastolic blood pressures were taken during the NHANES physical examination by certified examiners. We used the mean of systolic and diastolic blood pressure measurements in our analyses unless only one measurement was available. Elevated systolic blood pressure was defined as systolic blood pressure ≥ 140 mm Hg. Elevated diastolic blood pressure was defined as diastolic blood pressure ≥ 90 mm Hg. We did not evaluate clinical hypertension as an outcome, but instead included self-reported antihypertensive medication use as a separate covariate in regression models. We chose this analytical approach because people diagnosed with hypertension who are taking medication may exhibit different health behaviors from those who have undiagnosed and/or untreated elevated blood pressure which in turn, may affect lead, allostatic load, and hypertension relationships. In our study population, antihypertensive medication users had significantly lower serum cotinine and alcohol consumption levels than people with elevated blood pressure who were not on medication (data not shown).

### Blood lead measurements

Blood samples were obtained by venipuncture during the physical examination, and lead concentrations were quantified using inductively coupled plasma mass spectrometry (CDC National Center for Environmental Health, Atlanta, Georgia). Twelve participants (<1%) had blood lead concentrations below the limit of detection (LOD) (0.30 μg/dL) which were substituted by the CDC with LOD $\sqrt{2}$.

### Allostatic load

Informed by prior studies [37,38] as well as data availability within NHANES, we operationalized AL by creating a cumulative index of physiologic dysregulation of the cardiovascular, inflammatory, and endocrine systems using the following seven biological markers: urinary creatinine, serum albumin, urinary C-reactive proteins (biomarkers of inflammation and immune response), plasma levels of glycosolated hemoglobin, serum triglycerides, HDL serum cholesterol (metabolic and cardiovascular biomarkers), and waist circumference (anthropometric biomarker). Cut-points were empirically defined using sex-specific quintiles for each of the seven components. A value of one was assigned to the lowest quintile and a value of five was assigned to the highest quintile. The highest quintile always corresponded to the highest risk category (e.g. large waist circumference, low creatinine clearance). We calculated the AL score by summing each participant’s rankings for the seven components; the AL score ranged from seven to 35.

### Statistical analyses

We calculated the mean and standard error (SE) for normally distributed variables such as systolic and diastolic blood pressures, and geometric mean (GM) and geometric standard error (GSE) for lognormally distributed variables such as lead and cotinine. In bivariate analyses, differences across groups were evaluated using the chi square test for categorical data and analysis of variance for continuous data.

We examined the association between lead exposure and blood pressure in several ways. In our primary analysis, we modeled systolic and diastolic blood pressure as dichotomous variables using logistic regression. To allow for potential non-linear relationships, we categorized lead exposure into quintiles and compared quintiles two, three, four, and five to the lowest quintile. Quintile cutoffs were based on the weighted distribution of lead. We also modeled both the blood pressure and lead exposure as continuous variables. In these models, blood lead concentrations were natural log transformed prior to construction of multivariate models.

In addition to blood lead, the model included the following biologic and social determinants of hypertension: age (continuous), race/ethnicity (non-Hispanic white, non-Hispanic black, Mexican American, other Hispanic, or other race), educational attainment [less than 12th grade (no diploma), high school graduate, some college/associates (AA) degree, or college graduate and above], and sex. We adjusted all models for self-reported use of antihypertensive medication (yes/no). The following potential confounders were also considered: marital status (married/living with partner, divorced/separated/widowed, or never married), smoking status (never, former, current), and alcohol consumption (never, < 1 drink per week, 1-3 drinks per week, or > 3 drinks per week). These covariates were retained in the final model if they were statistically significant or if they changed the beta coefficient for lead exposure by 10% or more.

To assess for potential effect modification by AL, we stratified the final multivariate model of blood lead and elevated blood pressure by AL status divided at the median AL score. To formally test the interactions observed in our stratified models, we included a lead by allostatic load interaction term in our full model along with cross-product terms of AL with all covariates in the final model (e.g. AL*sex, AL*age, etc.). To assess the impact of our definition of “high AL”, we conducted a sensitivity analysis where high AL was defined as an AL score above the 80th percentile. Lastly, to evaluate the impact of antihypertensive medication users on our results, we conducted a sensitivity analysis by removing antihypertensive medication users (n = 1640) from the final multivariate models.

All analyses were conducted in SUDAAN 10.0 (Research Triangle Institute, Cary, NC) and SAS 9.2 (SAS Institute Inc., Cary, NC). SUDAAN calculates variance estimates after incorporating the non-random sampling design and the sample population weights, which account for the unequal probability of selection into the survey and the oversampling of certain subgroups. Since we combined five cycles of data, we calculated new sample weights for each study participant according to the NHANES analytical guidelines [39]. For participants surveyed from 1999-2002, sample weights were equal to two-fifths of the four year sample weights. For participants surveyed from 2003-2008, sample weights were equal to one fifth of the two year sample weights. Statistical tests for trends of categorical variables were conducted by coding quintile categories as integers and evaluating tests for significance on the slope of the regression line. A (two-sided) *P*-value < 0.05 was considered significant and < 0.10 marginally significant.

## Results

### Participant characteristics

We excluded participants who were missing data or were pregnant (N = 1724), leaving a total of 8194 participants for our analyses. There were no significant differences in mean levels of blood lead concentration, blood pressure, or in the frequency of blood pressure medication use between the eligible and excluded groups. However, the excluded group had a higher prevalence of systolic and diastolic hypertension and a lower mean allostatic load score. Those not included were also more likely to be younger, female, non-Hispanic black or other/mixed race, less educated, never married, current or former smokers, and less likely to regularly drink alcohol (data not shown).

In our final study sample, blood lead levels in the highest lead quintile were approximately five times higher than those in the lowest lead quintile (GM (GSE) = 3.88 (0.03) μg/dL versus 0.76 (0.01) μg/dL). Participants in the higher lead quintiles were more likely to be older, male, non-Hispanic black, less educated, current smokers, and regular drinkers (Table 1). Systolic and diastolic blood pressures increased positively by blood lead quintile (*P* < 0.01) while use of antihypertensive medication was similar across lead quintiles (Table 1).

**Table 1:** **Descriptive characteristics for adults aged 40 to 65 years (N = 8194) in the national health and nutritional examination survey, United States, 1999-2008**^**a**^

		**Blood lead quintile**					
**Characteristic**	**Total**	**Quintile 1**	**Quintile 2**	**Quintile 3**	**Quintile 4**	**Quintile 5**	
	**(N = 8194)**	**(N = 1411)**	**(N = 1510)**	**(N = 1608)**	**(N = 1638)**	**(N = 2027)**	
Blood lead (μg/dL); geometric mean (GSE)	1.69 (0.02)	0.76 (0.01)	1.25 (0.00)	1.67 (0.00)	2.25 (0.01)	3.88 (0.03)	**
Systolic blood pressure (mm Hg); mean (SE)	124.33 (0.30)	122.23 (0.55)	122.43 (0.53)	124.59 (0.49)	125.48 (0.57)	126.92 (0.62)	**
Diastolic blood pressure (mm Hg); mean (SE)	74.81 (0.20)	74.30 (0.37)	74.16 (0.38)	74.99 (0.32)	74.96 (0.32)	75.65 (0.34)	**
Elevated blood pressure, % (SE)							
Systolic > 140 mm Hg	16.4 (0.6)	13.9 (1.2)	13.3 (1.1)	15.9 (1.2)	17.5 (1.0)	21.4 (1.4)	**
Diastolic > 90 mm Hg	7.8 (0.5)	5.5 (0.7)	6.5 (0.8)	8.1 (0.9)	9.2 (0.9)	9.6 (0.8)	**
Age (years), mean (SE)	50.9 (0.15)	48.4 (0.22)	50.0 (0.25)	51.3 (0.27)	52.2 (0.26)	52.6 (0.21)	**
Male sex, % (SE)	49.1 (0.6)	25.1 (1.7)	39.3 (1.5)	51.8 (1.4)	59.0 (1.5)	70.5 (1.5)	**
Race/Ethnicity, % (SE)							**
Non-Hispanic White	75.7 (1.3)	80.1 (1.6)	77.9 (1.7)	77.6 (1.4)	74.5 (1.6)	68.3 (2.1)	
Non-Hispanic Black	9.7 (0.8)	6.7 (0.7)	8.3 (0.8)	8.8 (0.9)	9.9 (0.9)	14.8 (1.4)	
Mexican American	5.7 (0.6)	5.3 (0.7)	5.2 (0.6)	5.5 (0.7)	5.4 (0.6)	7.2 (0.9)	
Other Hispanic	4.2 (0.6)	4.0 (0.7)	4.6 (1.1)	4.4 (0.9)	4.1 (0.7)	4.1 (0.9)	
Other	4.7 (0.4)	3.9 (0.7)	4.0 (0.7)	3.7 (0.6)	6.1 (0.9)	5.6 (0.8)	
Educational attainment, % (SE)							**
Less than 9th grade	5.4 (0.4)	3.2 (0.4)	4.2 (0.5)	5.7 (0.7)	5.6 (0.6)	8.3 (0.7)	
9th - 12th grade (no diploma)	10.9 (0.5)	7.4 (0.9)	9.1 (0.8)	8.7 (0.9)	12.1 (0.9)	17.3 (1.2)	
High school graduate or equivalent	24.6 (0.8)	22.7(1.5)	22.9 (1.5)	24.6 (1.4)	26.0 (1.5)	26.7 (1.4)	
Some college or associates (AA) degree	30.6 (0.8)	32.0 (1.5)	30.3 (1.7)	32.1 (1.3)	29.5 (1.6)	29.3 (1.1)	
College graduate or above	28.5 (1.2)	34.7 (2.0)	33.5 (2.0)	28.9 (1.9)	26.9 (1.7)	18.3 (1.4)	
Marital status, % (SE)							**
Married or living with partner	73.2 (0.8)	75.6 (1.6)	75.6 (1.2)	72.6 (1.4)	71.4 (1.5)	70.8 (1.4)	
Divorced, separated, or widowed	19.9 (0.7)	17.5 (1.4)	18.3 (1.2)	20.0 (1.2)	22.2 (1.4)	21.6 (1.3)	
Never married	6.9 (0.4)	6.9 (0.8)	6.1 (0.8)	7.4 (0.9)	6.4 (0.7)	7.7 (0.8)	
Smoking status, % (SE)							**
Never	47.2 (0.8)	69.1 (1.5)	56.4 (1.6)	45.4 (1.7)	36.3 (1.6)	28.9 (1.3)	
Former	28.9 (0.7)	23.4 (1.3)	27.4 (1.5)	31.6 (1.4)	31.0 (1.3)	31.1 (1.4)	
Current	23.9 (0.8)	7.4 (0.8)	16.2 (1.1)	23.0 (1.5)	32.7 (1.8)	40.0 (1.4)	
Alcohol consumption, % (SE)							**
None	25.5 (1.0)	36.9 (2.0)	30.0 (1.6)	22.1 (1.3)	21.8 (1.3)	16.6 (1.2)	
Less than 1 drink per week	34.8 (0.9)	39.9 (1.7)	36.6 (1.5)	34.8 (1.6)	31.3 (1.4)	31.5 (1.6)	
1 to 3 drinks per week	11.4 (0.5)	12.6 (1.1)	13.0 (1.1)	13.0 (1.0)	10.4 (0.9)	8.1 (0.7)	
3 or more drinks per week	28.3 (0.9)	10.6 (1.2)	20.4 (1.3)	30.2 (1.7)	36.6 (1.5)	43.8 (1.7)	
Use of antihypertensive medication, % (SE)	23.5 (0.7)	26.3 (1.5)	22.4 (1.5)	21.9 (1.3)	23.1 (1.3)	23.7 (1.4)	

*Abbreviations:GSE* geometric standard error, *SE* standard error.

^a^ All estimates are adjusted for survey design and sample weights. Differences across groups were evaluated using the chi square test for categorical data and analysis of variance for continuous data. **P < 0. 05.

Distribution of blood lead quintiles varied by AL status, and elevated blood pressures were more prevalent in the high AL group (Table 2). Antihypertensive medication use was approximately twice as common in the high AL group compared to the low AL group. We observed a significant inverse relationship between educational attainment and AL, and AL varied by race/ethnicity with non-Hispanic Whites having the lowest AL score and Hispanics having the highest AL score. Differences between Hispanics (both Mexican American and other Hispanics) and non-Hispanic Whites were statistically significant (*P* < 0.001) while differences between non-Hispanic Whites and non-Hispanic Blacks were not (data not shown).

**Table 2:** Distribution of blood pressure measures, lead exposure, and allostatic load components by allostatic load score among adults aged 40 to 65 years in the national health and nutritional examination survey, United States, 1999-2008 (N = 8194)

**Variable**	**Low allostatic load**^**a **^**(N = 4069)**	**High allostatic load**^**a **^**(N = 4125)**
Blood pressure, % (SE)		
Elevated systolic blood pressure	12.8 (0.8)	20.7 (0.8)
Elevated diastolic blood pressure	6.9 (0.5)	8.8 (0.6)
Pb Exposure, % (SE)		
Quintile 1 (≤1.05 μg/dL)	17.5 (1.0)	22.9 (1.0)
Quintile 2 (1.06 - 1.44 μg/dL)	20.1 (0.9)	19.9 (0.8)
Quintile 3 (1.45 – 1.90 μg/dL)	20.7 (0.9)	19.3 (0.8)
Quintile 4 (1.91 – 2.69 μg/dL)	19.9 (0..7)	19.5 (0.9)
Quintile 5 (> 2.70 μg/dL)	21.8 (0.8)	18.5 (0.8)
Antihypertensive medication use, % (SE)	15.7 (0.7)	32.8 (0.9)
Components of Allostatic Load	mean ± SE (range)	
Triglycerides (mg/dL),	115.3 ± 1.56 (26 - 1000)	222.3 ± 4.02 (31 - 3854)
HDL cholesterol (mg/dL)	59.8 ± 0.35 (8 -164)	44.9 ± 0.27 (12 - 108)
Waist circumference (cm)	91.8 ± 0.23 (59.4 -157.1)	108.35 ± 0.32 (70.7 – 168.4)
C-reactive protein (mg/dL)	0.23 ± 0.01 (0.01 - 17.5)	0.66 ± 0.02 (0.01 – 18.5)
Creatinine (mg/dL)	123.66 ± 1.49 (7 – 609)	112.86 ± 1.61 (7 – 774)
Albumin (g/dL)	4.38 ± 0.01 (2.5 – 5.3)	4.16 ± 0.01 (2.3 - 5.3)
Glycosolated hemoglobin (%)	5.31 ± 0.01 (3.3 – 14.0)	5.95 ± 0.02 (4.1 – 18.8)
Composite allostatic load score	16.9 ± 0.02 (7 – 21)	25.7 ± 0.06 (22-35)

^a^Low allostatic load is equal to a score between 7 and 20. High allostatic load is equal to a score between 21 and 35. Elevated systolic BP defined as average BP ≥ 140 mm Hg and elevated diastolic BP defined as average BP ≥ 90 mm Hg.

### Lead, allostatic load, and elevated blood pressure

In linear regression models for blood pressure modeled continuously, lead exposure was a significant predictor of elevated diastolic but not systolic blood pressure after adjustment for covariates (Table 3). Diastolic blood pressure was significantly higher in the fourth and fifth lead exposure quintiles relative to the reference quintile, and there was evidence of a linear, dose-response relationship (*P* = 0.0001 for trend). When models were stratified by high versus low AL, the association between lead and systolic blood pressure was not significant in either of the AL groups. For diastolic blood pressure, we observed positive associations between lead exposure and diastolic blood pressure in both AL groups. The highest lead quintile had a larger effect on diastolic blood pressure among the high AL group (ß =2.01; 95% CI, 0.24, 3.79) than in the low AL group (ß = 1.79; 95% CI, 0.62, 2.95), although the lead by AL interaction term was not statistically significant (Table 3).

**Table 3:** **Adjusted difference in systolic and diastolic blood pressure by quintiles of blood lead exposure among adults aged 40 to 65 years in the national health and nutritional examination survey, United States, 1999-2008**^**a**^

	**All participants (N = 8,194)**		**Low allostatic load**^**b **^**(N = 4,069)**		**High allostatic load**^**b **^**(N = 4,125)**	
	**Difference**	**95% CI**	**Difference**	**95% CI**	**Difference**	**95% CI**
Systolic blood pressure (mm Hg)^c^						
Pb Exposure						
Quintile 1	0	Reference	0	Reference	0	Reference
Quintile 2	−0.79	−2.07, 0.49	−1.08	−2.40, 0.25	0.13	−2.17, 2.44
Quintile 3	0.24	−1.22, 1.71	−0.27	−2.09, 1.55	1.68	−0.65, 4.02
Quintile 4	0.20	−1.26, 1.67	0.20	−1.59, 1.98	0.97	−1.50, 3.44
Quintile 5	0.63	−1.07, 2.33	0.67	−1.24, 2.58	1.60	−0.62, 3.82
Test for trend	*P = 0.24*		*P = 0.21*		*P = 0.14*	
Diastolic blood pressure (mm Hg)^d^						
Pb Exposure						
Quintile 1	0	Reference	0	Reference	0	Reference
Quintile 2	0.08	−0.80, 0.97	−0.30	−1.60, 1.01	0.80	−0.51, 2.11
Quintile 3	0.99	0.16, 1.82	1.06	0.03, 2.09	1.23	−0.11, 2.57
Quintile 4	1.17	0.25, 2.08	1.36	0.11, 2.60	1.21	−0.21, 2.63
Quintile 5	1.76	0.75, 2.78	1.79	0.62, 2.95	2.01	0.24, 3.79
Test for trend	*P = 0.0001*		*P = 0.0002*		*P = 0.02*	

*Abbreviations:CI* confidence interval.

^a^Adjusted for age, sex, race/ethnicity, education, marital status, smoking status, alcohol consumption, and antihypertensive medication use.

^b^Low allostatic load is equal to a score between 7 and 20. High allostatic load is equal to a score between 21 and 35.

^c^Pb and AL test of interaction for systolic blood pressure: *P* = 0.75.

^d^Pb and AL test of interaction for diastolic blood pressure: *P* = 0.77.

The effect of lead was more apparent when blood pressure outcomes were modeled dichotomously using clinical cutoffs for elevated blood pressure (Table 4). There was evidence of a linear dose-response for quintiles of blood lead and elevated systolic blood pressure in the high AL group (p = 0.03) but not the low AL group (p = 0.24). Similarly, the relationship between lead exposure and elevated diastolic blood pressure was stronger among the high AL group than the low AL group. Within the high AL group, the fourth and fifth quintiles had significantly increased odds of elevated blood pressure compared to lowest quintile [OR = 1.92, (95% CI, 1.07, 3.47) and OR =2.28 (95% CI, 1.33, 3.91), respectively] and there was evidence of a linear, dose-response relationships (p _=_ 0.002). In the low AL group, none of the quintile effects were significantly different than the referent group although there was evidence of a linear trend (p =0.03). The lead by AL interaction term was not statistically significant for either systolic or diastolic blood pressure models.

**Table 4:** **Adjusted odds ratio for elevated systolic and diastolic blood pressure by quintiles of blood lead exposure among adults aged 40 to 65 years in the national health and nutritional examination survey, United States, 1999-2008**^**a**^

	**All participants (N = 8,194)**		**Low allostatic load**^**b **^**(N = 4,069)**		**High allostatic load**^**b **^**(N = 4,125)**	
	**OR**	**95% CI**	**OR**	**95% CI**	**OR**	**95% CI**
Elevated systolic blood pressure (≥ 140 mm Hg) ^c^						
Pb Exposure						
Quintile 1	1.00	Reference	1.00	Reference	1.00	Reference
Quintile 2	0.87	0.66, 1.15	0.81	0.55, 1.19	0.96	0.66, 1.38
Quintile 3	1.00	0.76, 1.31	0.86	0.61, 1.21	1.20	0.82, 1.75
Quintile 4	1.03	0.78, 1.37	0.88	0.56, 1.39	1.23	0.86, 1.76
Quintile 5	1.23	0.92, 1.65	1.14	0.79, 1.66	1.40	0.99, 1.97
Test for trend	*P = 0.06*		*P = 0.24*		*P = 0.03*	
Elevated diastolic blood pressure (≥ 90 mm Hg) ^d^						
Pb Exposure						
Quintile 1	1.00	Reference	1.00	Reference	1.00	Reference
Quintile 2	1.22	0.82, 1.81	0.85	0.45, 1.60	1.66	0.93, 2.95
Quintile 3	1.56	1.11, 2.19	1.51	0.83, 2.75	1.67	0.97, 2.85
Quintile 4	1.80	1.24, 2.60	1.73	0.96, 3.12	1.92	1.07, 3.47
Quintile 5	1.77	1.25, 2.50	1.46	0.80, 2.68	2.28	1.33, 3.91
Test for trend	*P = 0.0002*		*P = 0.03*		*P = 0.002*	

*Abbreviations:CI* confidence interval, *OR* odds ratio.

^a^Adjusted for age, sex, race/ethnicity, education, marital status, smoking status, alcohol consumption, and antihypertensive medication use.

^b^Low allostatic load is equal to a score between 7 and 20. High allostatic load is equal to a score between 21 and 35.

^c^Pb and AL test of interaction for elevated systolic blood pressure: *P* = 0.74.

^d^Pb and AL test of interaction for elevated diastolic blood pressure: *P* = 0.40.

**Figure 1:**
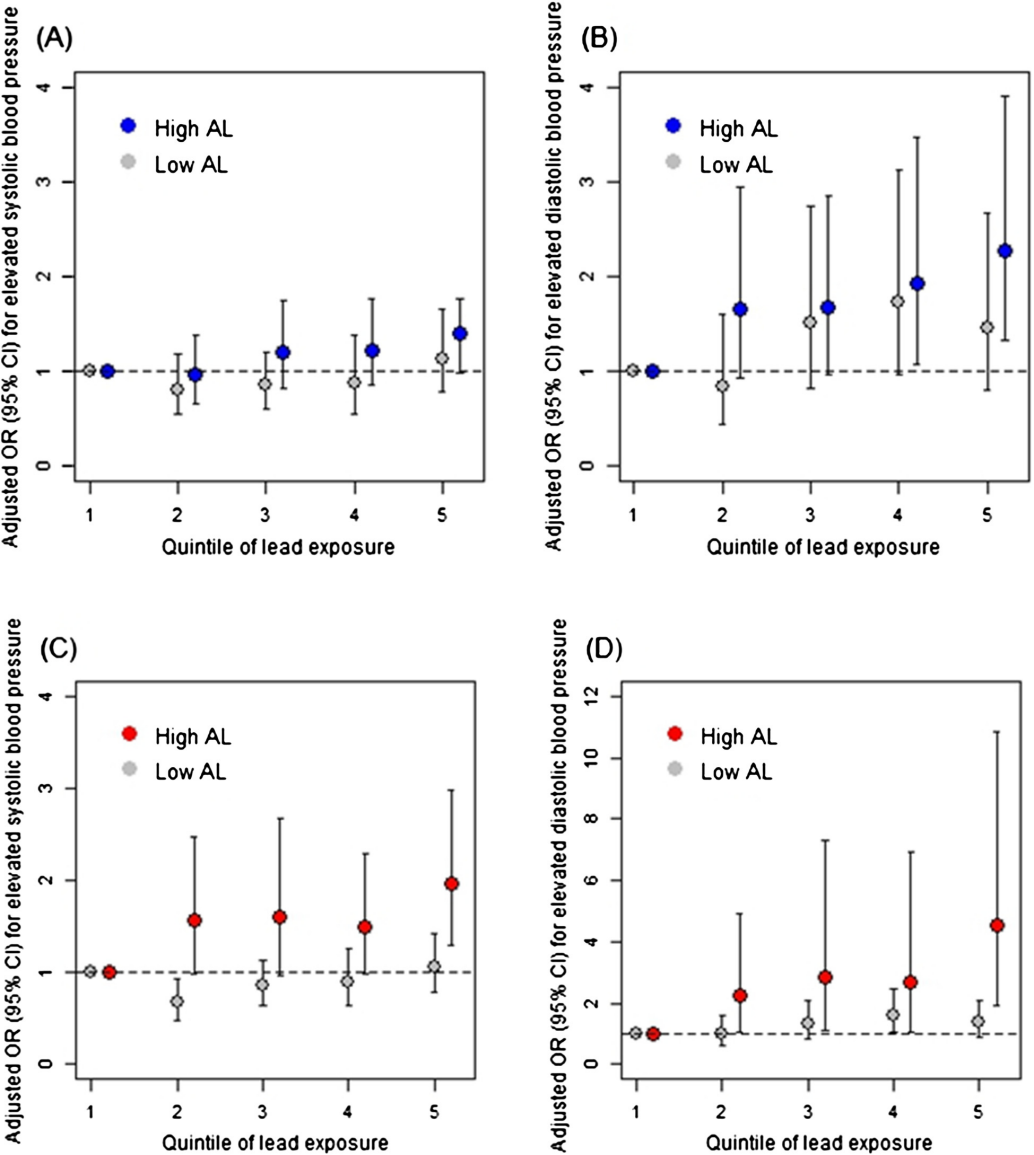
**The association between quintile of blood lead exposure and odds of elevated systolic and diastolic blood pressure for those with high allostatic load (high AL) versus those with low allostatic load (low AL) in the National Health and Nutritional Examination Survey, United States, 1999-2008.** All figures are adjusted for age, age squared, sex, race/ethnicity, educational attainment, marital status, cotinine, alcohol consumption, and anti-hypertensive medication. In Figures **A** and **B**, high AL is defined as the top 50th percentile whereas in Figures **C** and **D**, high AL is defined as the top 20th percentile.

We also conducted several sensitivity analyses to examine the impact of model selection and AL definitions on the results. A more extreme definition for high AL (i.e., the top 80%), rendered larger differences in effect estimates for lead exposure between low and high AL groups (Figure 1). However, these estimates also had wider confidence intervals and the lead by AL interaction terms were not statistically significant. We also examined effects of lead exposure modeled continuously. These results, which are presented in Additional file 1: Tables S1 and Additional file 2: Table S2, were generally similar to models in which lead exposure was modeled in quintiles.

After removing antihypertensive medication users from the analysis (Additional file 3: Tables S3 and Additional file 4: Table S4), effect estimates for lead exposure on differences in systolic blood pressure remained null in both groups. However, in logistic regression models, lead effects on odds of elevated systolic blood pressure in the highest quintile were more pronounced in the high AL group (OR = 1.75, (95% CI: 1.09, 2.83) compared to the low AL group (OR = 1.08, (95% CI: 0.63, 1.84). Effect estimates for lead exposure on differences in diastolic blood pressure in this restricted model were similar to those obtained in the full model. However, lead effects on the odds of elevated diastolic blood pressure were attenuated in both groups, The odds ratio for the fifth quintile relative to the reference quintile was 1.33 (95% CI: 0.65, 2.73) and 1.82 (95% CI: 0.91, 3.64), in the low and high AL group, respectively, and the linear dose response trends in either group were no longer statistically significant.

## Discussion

### Interpretation of the results and underlying mechanisms

In this cross-sectional study of a nationally representative population of U.S. middle-aged adults, we found slightly higher effect estimates of low-level lead exposure on the risk of elevated blood pressure among participants with relatively higher AL. Higher blood lead levels were significantly associated with increased odds of elevated systolic and diastolic blood pressure among adults with high AL, a biologic indicator of chronic stress response, while lead effects on blood pressure were less pronounced and not significant for those in the low AL group. P-values for multiplicative interactions between lead and AL were not significant. Although this is the first examination of whether higher AL may amplify vulnerability to the adverse effects of lead exposure, our findings are consistent with interactions between lead and psychosocial stressors observed in animals [40], and in human studies [34,41,42]. Lead and chronic stress may impact several common biological pathways which could, in turn, affect blood pressure regulation. Most importantly, both chronic stress and lead can influence the HPA axis, which can promote inflammation, vasoconstriction, and adrenergic activity. Lead and chronic stress can also increase the level of reactive oxygen species leading to oxidative stress [18,22,43]. Stronger effects of lead exposure on blood pressure among participants with higher AL may be a consequence of the direct effect of lead on components of the physiological stress response system underlying the AL measure. For example, previous studies have found associations between blood lead and general distress [44] and bone lead and phobic anxiety [45]. However, in our study, the distribution of lead exposure was similar between participants with high versus low AL. Moreover, we observed significant dose-response relationships between lead and blood pressure among the high AL group, particularly for the diastolic blood pressure outcome. The consistency of this stronger effect across different lead levels and health outcomes suggests that AL may in fact amplify the blood lead and blood pressure relationship.

### Comparison with other studies

Our study supports a growing body of epidemiological evidence indicating that physiologic dysregulation due to chronic stress may amplify observed associations between environmental pollutant exposures and adverse health outcomes [4,5,8,23,46]. Peters et al. [34] examined the modifying effects of self-reported stress on the relationship between bone lead and hypertension in a longitudinal cohort of older white men from the greater Boston area. They found that men with higher bone lead concentrations and higher levels of stress are at increased risk of developing hypertension than those with lower stress levels. In cross-sectional analyses of subjects without hypertension at baseline, they found an interactive effect between lead and stress for systolic blood pressure but not diastolic blood pressure. In this study, we observed stronger lead effects by AL on diastolic blood pressure for various model specifications. Among the high AL group in the main analyses (Table 4), effect estimates of lead were larger for risk of elevated diastolic blood pressure than systolic blood pressure. When participants taking hypertensive medications were removed in the sensitivity analysis (Additional file 3: Table S3 and Additional file 4: Table S4), stronger effects were still observed among the higher AL group for diastolic blood pressure in the linear regression models, but these effects were non-significant in the logistic models (Additional file 4: Table S4).

Results of most previous studies report small but significant effects of lead exposure on both systolic and diastolic blood pressure [15,16]. Some studies report stronger effects on diastolic blood pressure whereas in other studies the reverse is true [16]. These inconsistencies may result from differences in ranges of lead exposures, age of study participants, measurement error associated with blood pressure determination, and covariates included in the analysis. For example, systolic blood pressure increases linearly with age, whereas diastolic blood pressure increases with age up to 55 and then declines [7]. Therefore, elevated diastolic blood pressure may be more common among our study population of middle-aged adults than studies with older populations. Indeed, our results showing lead exposure effects on diastolic blood pressure across all participants (Tables 3 and 4) are consistent with those from another study that examined effects of blood lead levels on elevated blood pressure among women aged 40-59 years from the NHANES III survey [47].

### Strengths and limitations

We had a large, heterogeneous study population which enhanced our ability to detect effects of lead exposure on blood pressure among participants with relatively higher AL. The racial and economic diversity provided by the NHANES sampling strategy is particularly useful for this analysis since lead exposures and AL scores were both socioeconomically patterned. Moreover, we were able to control for a number of potential confounders including: age, educational attainment, race/ethnicity, smoking, alcohol consumption, marital status, and antihypertensive medication use. However, residual confounding remains possible. In addition, the cross-sectional design of our study precludes a systemic assessment of the temporality of lead exposure and allostatic load, the potential for reverse causation between hypertension and AL, or the potential effects of cumulative lead exposures throughout the life course, since blood lead (as opposed to bone lead) mostly reflects recent and ongoing exposures as well as lead that has been mobilized from tissue stores such as bone [48]. Nevertheless, although our study could not address these limitations, we did systematically evaluate the robustness of our findings with different model specifications, and our results generally persisted in sensitivity analyses.

Most human health studies analyzing whether chronic stress exposure amplifies associations between pollutant exposures and adverse health outcomes have relied on self-assessments of chronic stress exposure, or individual and area-level SES metrics as proxies for stress exposure [23,34,41,49,50]. This is the first study to assess whether physiologic dysregulation as measured by allostatic load, amplifies vulnerability to the adverse health effects of toxicant exposures. Importantly, we used the NHANES data set that did not include the primary mediators of HPA axis activity (e.g. cortisol, epinephrine, norepinephrine, dehydroepiandrosterone sulfate (DHEA-S)), and instead utilized biomarkers of secondary effects of the chronic stress response. Findings from previous studies using similar AL algorithms with NHANES data suggest that the AL metric is useful in describing the biological risks associated with being socially disadvantaged. For example, AL scores are higher among socially marginalized groups [51,52] and AL attenuates the social gradients in ischaemic heart disease and periodontal disease [38]. Although alternate approaches to measuring AL using different metrics or applying different additive and weighting approaches have been considered [29], research indicates that no one approach has consistently stronger predictive value for different health outcomes [26,36,53], particularly cardiovascular effects. Instead, AL is a cumulative index that characterizes the combined effects of small, subclinical changes in several physiological systems [54]. The advantage of the AL score is that it provides an integrated, biological measure of physiologic dysregulation due to chronic stress response, although it does not elucidate the dominant sources of chronic stress in the study population. Despite the methodological challenges of understanding the extent to which AL is a direct indicator of stress exposure, future research should utilize biological measures of stress response in conjunction with subjective assessments of stress exposure and objective metrics of individual and area-level SES that may act as proxy indicators of social stressors (e.g. neighborhood-level poverty rate and individual perception of community social standing) [10,55]. This approach could enhance understanding about the modifying potential of different chronic stressors on pollutant exposures and adverse health outcome relationships.

## Conclusions

Our finding that AL may amplify the effect of blood lead on blood pressure suggests the need for more research to better understand the relevant biological and psychosocial pathways through which chemical exposures may differently affect the health of vulnerable populations, a significant portion of whom are disproportionately exposed to chronic psychosocial stressors (e.g. material deprivation, exposure to violence, lack of nutrition or access to health care). Cohort studies can expand exploration of mediating biological pathways, improve assessment of temporal issues, and facilitate further examination of interactive effects of environmental and social stress on other intrinsically susceptible populations (e.g. children) for other adverse health outcomes such as cognitive development, which has been linked to both chronic stress and lead exposure [40]. Most important, these results suggest that the “double jeopardy” of environmental and social stressors needs to be more systematically integrated into regulatory science and decision-making in order to better protect the health of vulnerable populations who often face disproportionate and elevated exposures to multiple chemical and non-chemical hazards.

## Abbreviations

AL: Allostatic load; CDC: Centers for disease control and prevention; CI: Confidence interval; HPA: Hypothalamic–pituitary–adrenal; LOD: Limit of detection; NHANES: National health and nutritional examination survey; OR: Odds ratio; SES: Socioeconomic status; SE: Standard error.

## Competing interests

All authors declare they have no actual or potential competing financial interests.

## Authors’ contributions

RMF and EDS conceived of the study, and ARZ and RMF further specified the research question with RMF directing the study’s implementation. RMF and EDS oversaw the statistical analysis and ARZ conducted all of the programming and data analysis. ARZ and RMF led the writing with significant input from EDS. All authors read and approved the final manuscript.

## Supplementary Material

Additional file 1: Table S1 Adjusted difference in systolic and diastolic blood pressure by blood lead exposure among adults aged 40 to 65 years in the National Health and Nutritional Examination Survey, United States, 1999-2008^a^. Click here for file

Additional file 2: Table S2 Adjusted odds ratio for elevated systolic and diastolic blood pressure by blood lead exposure among adults aged 40 to 65 years in the National Health and Nutritional Examination Survey, United States, 1999-2008^a^. Click here for file

Additional file 3: Table S3 Adjusted difference in systolic and diastolic blood pressure by quintiles of blood lead exposure among adults aged 40 to 65 years in the National Health and Nutritional Examination Survey, United States, 1999-2008^a^, excluding those on antihypertensive medication. Click here for file

Additional file 4: Table S4 Adjusted odds ratio for elevated systolic and diastolic blood pressure by quintiles of blood lead exposure among adults aged 40 to 65 years in the National Health and Nutritional Examination Survey, United States, 1999-2008^a^, excluding those on antihypertensive medication. Click here for file
