# Supplementary material for: Allostatic load amplifies the effect of blood lead levels on elevated blood pressure among middle-aged U.S. adults: a cross-sectional study
Source: Environ Health. 2013 Aug 16;12:64. doi: 10.1186/1476-069X-12-64 (PMC3847858; doi:10.1186/1476-069X-12-64)
Supplement: Additional file 4: Table S4 — Adjusted odds ratio for elevated systolic and diastolic blood pressure by quintiles of blood lead exposure among adults aged 40 to 65 years in the National Health and Nutritional Examination Survey, United States, 1999-2008a, excluding those on antihypertensive medication. [file 1476-069X-12-64-S4.doc]

Table S4. Adjusted odds ratio for elevated systolic and diastolic blood pressure by quintiles of blood lead exposure among adults aged 40 to 65 years in the National Health and Nutritional Examination Survey, United States, 1999-2008^a^, excluding those on antihypertensive medication.

|  | **All Participants**  (N=6,011) | |  | **Low Allostatic Load^b^**  (N=3,352) | |  | **High Allostatic Load^b^**  (N=2,659) | |
| --- | --- | --- | --- | --- | --- | --- | --- | --- |
|  | OR | 95 % CI |  | OR | 95 % CI |  | OR | 95 % CI |
| Elevated systolic blood pressure(≥ 140 mm Hg)^c^ | | | | | | | | |
| Pb Exposure |  |  |  |  |  |  |  |  |
| Quintile 1 | 1.00 | Reference |  | 1.00 | Reference |  | 1.00 | Reference |
| Quintile 2 | 0.91 | 0.65, 1.27 |  | 0.82 | 0.54, 1.23 |  | 1.09 | 0.66, 1.80 |
| Quintile 3 | 1.08 | 0.74, 1.57 |  | 0.86 | 0.56, 1.33 |  | 1.45 | 0.84, 2.49 |
| Quintile 4 | 1.08 | 0.75, 1.55 |  | 0.86 | 0.49, 1.50 |  | 1.46 | 0.91, 2.34 |
| Quintile 5 | 1.32 | 0.87, 1.98 |  | 1.08 | 0.63, 1.84 |  | 1.75 | 1.09, 2.83 |
| Test for Trend | *P = 0.09* | |  | *P = 0.56* | |  | *P = 0.009* | |
| Elevated diastolic blood pressure(≥ 90 mm Hg)^d^ | | | | | | | | |
| Pb Exposure |  |  |  |  |  |  |  |  |
| Quintile 1 | 1.00 | Reference |  | 1.00 | Reference |  | 1.00 | Reference |
| Quintile 2 | 1.06 | 0.68, 1.65 |  | 0.77 | 0.38, 1.56 |  | 1.63 | 0.83, 3.20 |
| Quintile 3 | 1.43 | 0.92, 2.23 |  | 1.53 | 0.75, 3.11 |  | 1.39 | 0.76, 2.54 |
| Quintile 4 | 1.48 | 0.92, 2.37 |  | 1.54 | 0.78, 3.04 |  | 1.51 | 0.76, 3.01 |
| Quintile 5 | 1.47 | 0.92, 2.34 |  | 1.33 | 0.65, 2.73 |  | 1.82 | 0.91, 3.64 |
| Test for Trend | *P = 0.05* | |  | *P = 0.13* | |  | *P = 0.16* | |

Abbreviations: CI, confidence interval; OR, odds ratio

^a^Adjusted for age, sex, race/ethnicity, education, marital status, smoking status and alcohol consumption.

^b^Low allostatic load is equal to a score between 7 and 20. High allostatic load is equal to a score between 21and 35.

^c^Pb and AL test of interaction for elevated systolic blood pressure: *P*=0.50

^d^Pb and AL test of interaction for elevated diastolic blood pressure: *P*=0.36
